# Effect of Conscious Sedation vs. General Anesthesia on Outcomes in Patients Undergoing Mechanical Thrombectomy for Acute Ischemic Stroke: A Prospective Randomized Clinical Trial

**DOI:** 10.3389/fneur.2020.00170

**Published:** 2020-03-24

**Authors:** Chunguang Ren, Guangjun Xu, Yanchao Liu, Guoying Liu, Jinping Wang, Jian Gao

**Affiliations:** Department of Anaesthesiology, Liaocheng People's Hospital, Liaocheng, China

**Keywords:** conscious sedation, general anesthesia, mechanical thrombectomy, acute ischemic stroke, dexmedetomidine

## Abstract

**Background:** Although several studies have compared conscious sedation (CS) with general anesthesia (GA) in patients undergoing mechanical thrombectomy (MT), there has been no affirmative conclusion. We conducted this trial to assess whether CS is superior to GA for patients undergoing MT for acute ischemic stroke (AIS).

**Methods:** Acute ischemic stroke patients with anterior circulation large vascular occlusion were randomized into two groups. The primary outcome was modified Rankin scale score (0–2) at 90 days after stroke. Secondary outcomes included intraprocedural hemodynamics, time metrics, successful recanalization, neurointerventionalist satisfaction score, National Institutes of Health Stroke Scale (NIHSS) score, and Alberta Stroke Program Early CT Score (ASPECTS) at 48 h post-intervention, mortality at discharge and 3 months after stroke, and complications.

**Results:** Compared with the CS group, heart rate was significantly lower at T1–T8 in the GA group except at T4 (*P* < 0.05). Mean arterial pressure (MAP) and systolic blood pressure were significantly lower in the GA group at T4–T6 and T9 (*P* < 0.05). Pulse oxygen saturation was significantly higher at T2–T9 in the GA group (*P* < 0.05). There were no significant differences in time metrics, vasoactive drug use, occurrence of >20% fall in MAP, pre-recanalization time spent with >20% fall in MAP, neurointerventionalist satisfaction, successful recanalization rate, NIHSS, and ASPECTS scores at 48 h post-intervention, and mortality rate at discharge and 3 months after stroke (*P* > 0.05). The cerebral infarction rate at 30 days was greater in the CS group, but not significantly (*P* > 0.05). There were no differences in complication rates except for pneumonia (*P* > 0.05). Conversion rate from CS to GA was 9.52%.

**Conclusion:** Anesthetic management with GA or CS during MT had no differential impact on the functional outcomes and mortality at discharge or 3 months after stroke in AIS patients, but CS led to more stable hemodynamics and lower incidence of pneumonia.

## Introduction

Acute ischemic stroke (AIS) is a leading cause of death and disability in China (1). A typical AIS patient loses approximately 1.9 million neurons each minute if not treated promptly (2). As a result, treatment and prevention of stroke are a major healthcare issue throughout the world (3). Efforts are currently being made to optimize medical services associated with AIS. Time from stroke onset to recanalization is crucial for salvaging the ischemic penumbra, improving the neurological outcomes, and decreasing morbidity and mortality (4–8). As a result, the primary goal of treatment in patients with AIS is to recanalize the brain as quickly and safely as possible (9). However, fewer than 33% of patients with large vascular occlusion (LVO) experience unsuccessful recanalization when only treated with intravenous recombinant tissue plasminogen activator, such as alteplase, within 4.5 h after stroke onset (10). Mechanical thrombectomy (MT) is an alternative to standard intravenous thrombolytic therapy for AIS patients who are disabled or who have other contraindications (such as recent surgery or coagulopathy) (11, 12). Recent randomized controlled trials have confirmed the efficacy of MT in patients with AIS due to anterior circulation LVO (13–16).

The goal of anesthesia management during intra-arterial treatment (IAT) for AIS is to increase patient comfort, facilitate treatment, reduce patient motion, and reduce the risk of complications (17). General anesthesia (GA), conscious sedation (CS), monitored anesthesia care (MAC), and local anesthesia (LA) are common anesthetic options during IAT. Unfortunately, most previous studies are single-center studies, have heterogeneous designs, and do not provide detailed descriptions of the anesthetic technique and pharmacologic details regarding endovascular treatment for AIS (18–20). Both GA and CS have their advantages and shortcomings. The benefits of GA include airway protection, pain control, patient immobility, and better radiographic imaging, whereas CS might be associated with less manpower and time, lower cost, fewer hemodynamic fluctuations, and ability to assess neurological function during the procedure (21). A retrospective study found that neurological outcomes were superior in patients who underwent CS compared with those who underwent GA (22). However, recently, randomized clinical trials have shown that GA leads to a higher rate of functional independence at 3 months after treatment (23–25). As a result, the Society for Neuroscience in Anesthesiology and Critical Care published the following consensus statement on anesthetic management of patients undergoing IAT for AIS; the choice of anesthetic technique and pharmacological agents should be individualized based on the clinical characteristics of each patient, tolerance of the procedure, and in close communication with the neurointerventionalist (26).

Dexmedetomidine (DEX) can be used for sedation, anxiolysis, analgesia, sympatholysis, and a reduced hemodynamic response. It can also reduce the requirement for both intravenous and inhalational anesthetics during surgery (27). As a result, DEX has been used in neurosurgery procedures such as awake craniotomy and deep brain stimulator implantation (28, 29). However, a recent study pointed out that DEX should be cautiously utilized in IAT, as hemodynamic instability and vasopressor requirement were significantly higher in the DEX group compared to the X group (30). There have been no recommendations on specific pharmacologic agents or anesthetic techniques for use during IAT for AIS. As a result, we performed this randomized clinical trial to compare the effects of CS vs. GA (both involving DEX) on the clinical and angiographic outcomes of patients undergoing MT for AIS.

## Methods

### Patients

Patients were recruited between August 2017 and December 2018 if they met the following criteria: American Society of Anesthesiologists (ASA) grades I–III; National Institutes of Health Stroke Scale (NIHSS) score < 20; AIS within 6.5 h of symptom onset; age ≥60 years; and intracranial proximal arterial occlusion in the anterior circulation (carotid artery, M1 or M2 segments of the middle cerebral artery, or A1 segment of the anterior cerebral artery) demonstrated by computed tomography angiography, magnetic resonance angiography, or digital subtraction angiography (DSA). We excluded patients with prestroke modified Rankin Scale (mRS) score > 2; hemorrhage demonstrated by computed tomography (CT); obvious or known difficult airway; cognitive impairment; disturbance of consciousness; hypoxemia (Spo_2_ < 90%); occlusion in the posterior circulation; or body mass index (BMI) >30 kg/m^2^. A computer-generated randomization table was used by an independent anesthesia assistant to allocate patients into two groups: the CS group (*n* = 42) and the GA group (*n* = 48).

### Anesthetic Management

Our anesthesia team included an attending anesthesiologist and an anesthesiologist assistant who were both blinded to group allocation. Standard ASA monitoring was employed. Blood pressure was routinely recorded non-invasively at 3-min intervals. During the procedure, supplemental oxygen (4 L/min) was delivered via a facemask in the CS group. The anesthetics used in the CS group were 1–1.5 mg/kg propofol as the loading dose followed by a maintenance dose of 2–4 mg/kg per hour propofol and 0.4–0.7 μg/kg per hour DEX titrated according to Richmond Agitation–Sedation Scale score of −2 to −3. Additionally, 1 μg/kg fentanyl or 0.04 mg/kg midazolam was used as a supplement. The GA group was induced with 1.5 mg/kg propofol, 2 μg/kg fentanyl, and 0.2 mg/kg cisatracurium after preoxygenation, and anesthesia was maintained with 4–6 mg/kg per hour propofol, 0.05–0.1 μg/kg per hour remifentanil, 0.2–0.4 μg/kg per hour DEX, and 0.1 mg/kg per hour cisatracurium. The anesthesiologist performed GA if the procedure was not possible due to restlessness of patients in the CS group. At the end of the surgery, recanalization was classified by the neuroradiologist according to the modified Thrombolysis in Myocardial Infarction (mTICI) perfusion grade. After removal of the tracheal intubation, all patients were transferred to the stroke unit or intensive care unit for at least 24 h and taken care of by an expert neurologist. Computed tomography or magnetic resonance imaging scans were generally obtained 8 h after treatment. Vasoactive drugs such as phenylephrine, ephedrine, atropine, urapidil, and nimodipine were used to keep blood pressure and heart rate fluctuation stable at the target values. Phenylephrine was the most commonly used vasopressor, and nimodipine was the most commonly used agent for hypotension.

### MT Procedure

A fellowship-trained neurointerventionalist performed the cerebrovascular angiography directly with 1% lidocaine at the access site. Mechanical thrombectomy was conducted by the same neurointerventionalist according to a previous study (31). Briefly, a 5F femoral sheath was introduced into the right femoral artery. After DSA confirmed the site of occlusion, a 6F or 8F femoral sheath was used to replace the 5F femoral sheath. A microcatheter was then placed in the artery distal to the thrombus, and the MT device was deployed distal to the thrombus. The MT device and microcatheter were removed through the guide catheter, and suction was applied during withdrawal. This process was repeated to ensure revascularization (defined as mTICI ≥ 2b) and to assess whether there were any complications. Adjunctive intra-arterial thrombolytics were also used during surgery. The thrombectomy approach (usage of a stent retriever or direct thrombus aspiration) was at the discretion of the neurointerventionalist, based on occlusion site, vascular status, and clot burden (32). In all cases, hemorrhagic events were systematically evaluated at the end of the procedure by CT.

### Outcome Measures

The primary outcome was a favorable neurologic outcome at 90 days (favorable outcome was defined as mRS score 0–2 and unfavorable as mRS score 3–6; 0–1, complete recovery; 2, mild disability; 3, moderate disability and transfer for rehabilitation; 4, transfer to nursing home with severe disability; 5–6, transfer to hospice/withdrawal of care) (33). Secondary outcomes included baseline characteristics, intraprocedural hemodynamics [recorded at the following time points: arrival at catheterization laboratory (T0); before puncture (T1); after angiography (T2); 3 min (T3), 6 min (T4), 9 min (T5), 12 min (T6), 15 min (T7), 30 min (T8), and 45 min (T9) during the procedure], successful recanalization (mTICI ≥ 2b; 0, no reperfusion; 1, penetration of affected vascular territory with minimal reperfusion; 2a, reperfusion of < 50% of the territory of the occluded vessel; 2b, reperfusion ≥50% but slower than expected filling of the territory of the occluded vessel; 3, complete reperfusion) (34), time metrics (time interval from stroke onset to catheterization laboratory, catheterization laboratory to groin puncture, and groin puncture to recanalization), vasopressor use, satisfaction score of the neurointerventionalist (10-point scale: 0, poorest; 10, excellent), complications (pneumonia, other infections, vessel perforation, vessel dissection, distal thrombus, and symptomatic intracerebral hemorrhage, defined as worsening involving NIHSS score ≥1 within 7 days after hemorrhage) (35), the conversion rate from CS to GA, Alberta Stroke Program Early CT Score (ASPECTS) and NIHSS score (0, no deficit; 42, most severe deficit) before and 48 h after intervention, and mortality at discharge and 3 months after stroke (36), The NIHSS score was evaluated by vascular neurology residents, and the mRS score was evaluated by stroke nurses. All of the investigators who assessed primary and secondary outcomes were blinded to group allocation.

### Statistical Analysis

Sample size was calculated to provide 80% power (at a two-sided significance level of 0.05) to detect a between-group difference in favorable neurologic outcome at 90 days of 20% (PASS 11.0; NCSS Statistical Software, Kaysville, UT, USA). This calculation indicated that 36 patients were required in each group. Assuming a dropout rate of 15%, at least 42 patients were recruited in each group.

Statistical analysis was performed with SPSS for Windows version 23.0 (SPSS Inc., Chicago, IL, USA). The Kolmogorov–Smirnov and Levene tests were used to assess data distribution and homogeneity of variance, respectively. Continuous data were expressed as mean and standard deviation or median and interquartile range. Between-group comparisons were performed using repeated-measures analysis of variance. The Kolmogorov–Smirnov *Z*-test was used for data that were not normally distributed. Categorical data were expressed as frequency and percentage and analyzed using χ^2^ tests or Fisher exact tests when appropriate. *P* < 0.05 was considered statistically significant.

## Results

### Baseline Data

**Figure 1:**
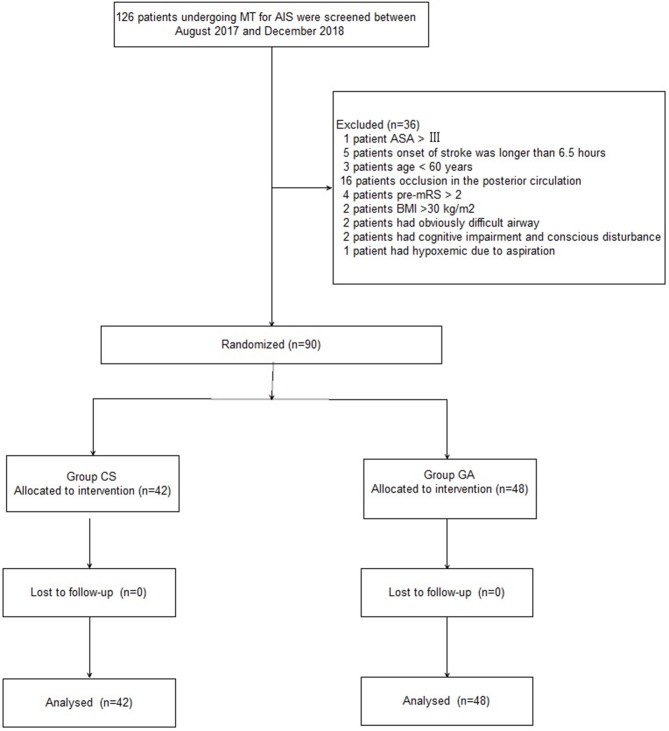
Patients enrollment flow diagram.

A Consolidated Standards of Reporting Trial (CONSORT) diagram showing the patient enrollment is displayed in Figure 1. Between August 2017 and December 2018, 126 patients undergoing MT for AIS were screened. A total of 36 patients were excluded because of the following reasons: occlusion in the posterior circulation (16 patients), stroke onset >6.5 h (five patients), preintervention mRS score > 2 (four patients), age < 60 years (three patients), BMI > 30 kg/m^2^ (two patients), obviously difficult airway (two patients), cognitive impairment and disturbance of consciousness (two patients), ASA grade > III (one patient), and hypoxemia due to aspiration (one patient). Ultimately, 90 patients were divided into two groups. The patients' baseline demographic, radiographic, and angiographic characteristics were comparable between the two groups (*P* > 0.05, Table 1).

**Table 1:** Baseline demographic, radiographic, and angiographic characteristics in two groups.

**Variable**	**Group CS (*n* = 42)**	**Group GA (*n* = 48)**	***P*-values**
Age (years)	69.19 ± 6.46	69.21 ± 5.78	0.989
Body weight (kg)	68.50 ± 9.25	67.65 ± 9.37	0.665
BMI (kg·m^−2^)	24.91 ± 2.59	23.84 ± 2.02	0.051
NIHSS	14.00 (11.00–16.00)	14.00 (11.00–16.00)	0.562
ASA I/II/III (*n*)	5/15/22	4/19/25	0.829
Sex (Male/Female)	24/18	26/22	0.777
rtPA, *n* (%)	34 (80.95%)	37 (77.08%)	0.797
Comorbidity, *n* (%)			0.969
Hypertension	20 (47.62%)	17 (35.42%)	
Diabetes	5 (11.90%)	6 (12.50%)	
Coronary heart disease	3 (7.14%)	3 (6.25%)	
Atrial Fibrillation	5 (11.90%)	4 (8.33%)	
Hyperlipidemia	2 (4.76%)	4 (8.33%)	
Previous stroke	5 (11.90%)	5 (10.42%)	
Prestroke mRS, *n* (%)			0.657
0	23 (47.62%)	21 (35.42%)	
1	13 (11.90%)	18 (12.50%)	
2	6 (7.14%)	9 (6.25%)	
Occluded segment, *n* (%)			0.978
M1	13 (30.95%)	15 (31.25%)	
M2	10 (23.81%)	13 (27.08%)	
ICA	16 (38.10%)	16 (33.33%)	
ACA	3 (7.14%)	4 (8.33%)	
ASPECTS, median (IQR)	9.00 (8.00–10.25)	9.00 (8.00–10.00)	0.446

*Variables presented as mean ± SD, median (interquartile range) or number of patients n (%). BMI, body mass index; NIHSS, National Institute of Health Stroke scale; ASA, American Society of Anesthesiology; rtPA, recombinant tissue plasminogen activator; mRS, modified Rankin Scale; ICA, internal carotid artery; ACA, anterior cerebral artery; ASPECTS, Alberta Stroke Program Early CT Score; GA, general anesthesia*.

### Procedural Data

**Figure 2:**
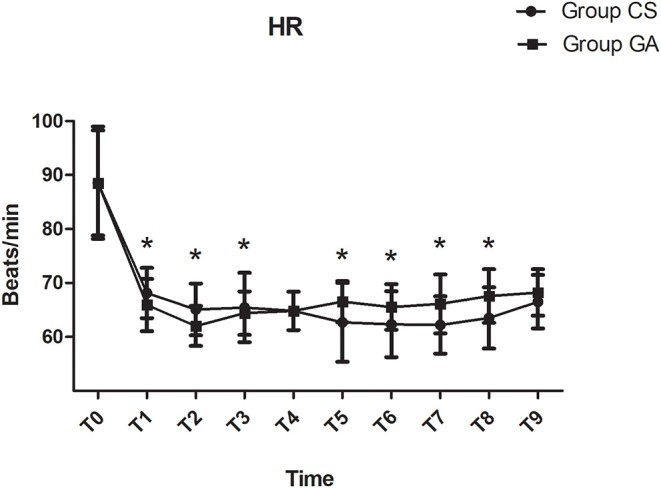
Heart rate changes during the procedure. *P < 0.05 vs. group CS.

**Figure 3:**
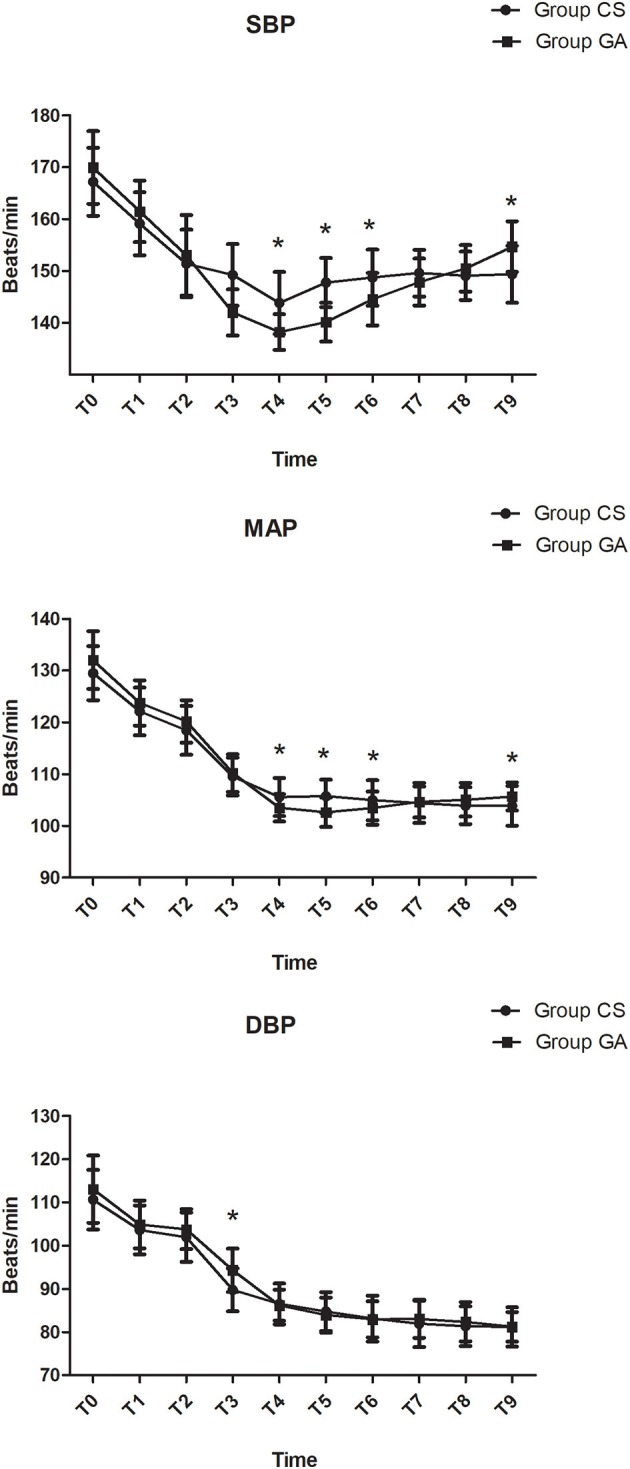
Hemodynamics changes during the procedure. *P < 0.05 vs. group CS.

**Figure 4:**
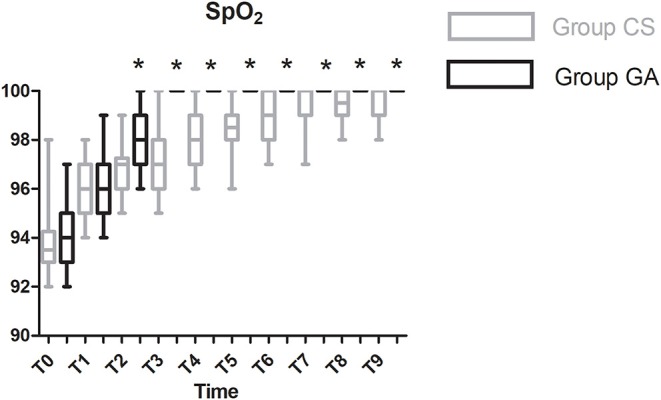
Spo_2_ changes during the procedure. *P < 0.05 vs. group CS.

Compared with the CS group, heart rate was significantly lower at T1–T8 in the GA group except at T4 (*P* < 0.05, Figure 2). Both mean arterial pressure (MAP) and systolic blood pressure were significantly lower in the GA group at T4–T6 and T9 (*P* < 0.05, Figure 3). However, there was no significant difference in diastolic blood pressure (DBP) between the two groups except at T3 (*P* > 0.05, Figure 3). Spo_2_ was significantly higher at T2–T9 in the GA group (*P* < 0.05, Figure 4).

Time metrics, comprising duration of surgery and anesthesia and time from stroke onset to catheterization laboratory, from catheterization laboratory to groin puncture, and from groin puncture to recanalization, were similar between the groups (*P* > 0.05, Table 2). Vasoactive drug use, occurrence of >20% fall in MAP, and time spent with >20% fall in MAP before recanalization were not significantly different between the two groups (*P* > 0.05, Tables 2, 3). The conversion rate from CS to GA was 9.52% (Table 2).

**Table 2:** Time intervals in the two groups.

**Variable**	**Group CS (*n* = 42)**	**Group GA (*n* = 48)**	***P*-values**
Duration of surgery (min)	161.24 ± 20.38	163.01 ± 21.67	0.676
Duration of anesthesia (min)	175.12 ± 20.57	176.85 ± 22.99	0.709
Occurrence of >20% fall in MAP compared with baseline, n (%)	22 (52.38%)	28 (58.33%)	0.672
Time spent with >20% fall in MAP compared with baseline (min)	9.00 (5.25–15.00)	9.00 (6.00–15.00)	0.926
Time from stroke onset to cath lab (min)	262.86 ± 62.29	247.38 ± 33.19	0.059
Time from cath lab to groin puncture (min)	11.45 ± 2.05	11.00 ± 1.64	0.248
Time from groin puncture to recanalization (min)	39.12 ± 11.86	46.98 ± 15.83	0.148
Convert to GA, *n* (%)	4 (9.52%)	**-**	**-**

*Variables presented as mean ± SD, median (interquartile range) or number of patients n (%). MAP, mean arterial pressure; GA, general anesthesia*.

**Table 3:** Consumption of vasoactive drugs during procedure in the two groups.

**Variable**	**Group CS (*n* = 42)**	**Group GA (*n* = 48)**	***P*-values**
Atropine	5 (11.90%)	5 (10.42%)	1.000
Ephedrine	7 (16.67%)	4 (8.33%)	0.335
Phenylephrine	7 (16.67%)	11 (22.92%)	0.599
Urapidil	7 (16.67%)	3 (6.25%)	0.179
Nimodipine	10 (23.81%)	5 (10.42%)	0.155

*Variables presented as number of patients n (%)*.

### Post-operative Data

The satisfaction of the neurointerventionalist, successful recanalization (mTICI ≥ 2b) rate, and NIHSS and ASPECTS scores at 48 h post-intervention were similar between the two groups (*P* > 0.05, Table 4). The mRS score and mortality rate at discharge and 3 months after stroke were not significantly different between the two groups (*P* > 0.05, Table 4). The cerebral infarction rate after 30 days was higher in the CS group; however, this difference was not significant (*P* > 0.05, Table 4).

**Table 4:** Consumption of post-operative variables in the two groups.

**Variable**	**Group CS (*n* = 42)**	**Group GA (*n* = 48)**	***P*-values**
Neurointerventionalist satisfaction score	9.00 (7.75–9.00)	9.00 (8.00–9.00)	0.388
Successful recanalization (mTICI ≥ 2b), *n* (%)	36 (85.71%)	42 (87.50%)	1.000
NIHSS at 48 h post-intervention	9.00 (7.00–11.25)	9.00 (7.00–11.00)	0.493
ASPECTS at 48 h post-intervention	12.00 (11.00–13.00)	12.00 (10.00–13.75)	0.076
Mortality, *n* (%)			
At discharge	5 (11.90%)	6 (12.50%)	1.000
3 months	9 (20.93%)	9 (18.75%)	0.796
mRS score, *n* (%)			
At discharge	2.00 (3.00–4.00)	2.00 (3.00–4.00)	0.890
3 months	2.50 (2.00–3.00)	2.50 (2.00–3.00)	0.652
Cerebral infarction after 30 d, *n*(%)	9 (20.93%)	11 (22.92%)	1.000

*Variables presented as median (interquartile range) or number of patients n (%). mTICI, modified Thrombolysis in Myocardial Infarction; NIHSS, National Institute of Health Stroke scale; mRS, modified Rankin Scale*.

The incidence of pneumonia was significantly higher in the GA group (*P* < 0.05, Table 5), but there were no significant differences between the two groups in complications such as vessel perforation, vessel dissection, distal thrombus, and other infections (*P* > 0.05, Table 5). Although there was a higher rate of symptomatic intracerebral hemorrhage in the GA group, this difference was also not significant (*P* > 0.05, Table 5).

**Table 5:** Post-operative adverse effects of patients in the two groups.

**Variable**	**Group CS (*n* = 42)**	**Group GA (*n* = 48)**	***P*-values**
Procedure-related complications	8 (19.05%)	9 (18.75%)	1.000
Symptomatic intracerebral hemorrhage	7 (16.7%)	9 (18.75%)	0.796
Pneumonia	2 (4.76%)	10 (20.83%)	0.031*
Other infections	2 (4.76%)	3 (6.25%)	1.000

Variables presented as number of patients n (%). *P < 0.05 vs. Group CS.

## Discussion

In this single-center study, we concluded that anesthetic management with GA or CS during MT for anterior circulation AIS had no differential impact on the functional outcomes or mortality rate at discharge or at 3 months after stroke, although there were more stable hemodynamics and a lower incidence of pneumonia in the CS group. There were no differences in recanalization rate, satisfaction of the neurointerventionalist, NIHSS and ASPECTS scores at 48 h post-intervention, time metrics, or most procedure-related complications between the two groups.

There has been an ongoing debate about the effects of the different anesthesia techniques during MT for AIS in recent years (18, 20). As early as 2008, a survey of 68 members of the Society of Vascular and Interventional Neurology (SVIN) showed that GA was the most commonly used method followed by CS, MAC, and LA (37). A previous study found that CS was associated with an increased mortality rate and poor functional outcomes compared with LA. Additionally, CS did not reduce either interventional complications or duration of intervention. As a result, authors suggested that CS had no advantage if LA can be safely implemented during IAT for AIS (38). A Nordic survey found that 84% of medical centers had institutional guidelines on anesthetic management, and 63% were able to provide a 24 h immediate response to an endovascular therapy request. Uncontrolled patient movements (82%) and loss of airway (35%) were still the most common reasons for converting to GA (39, 40). Besides, a previous study found that most LVOs in Caucasians are located in proximal blood vessels, and *in situ* thrombus is much more common in Asians, which is more difficult to recanalize (41). In our study, the rate of successful reperfusion and mortality rate at 3 months after stroke are inconsistent with the results of a previous systematic review and meta-analysis (21). This may be due to the intervention heterogeneity and use of thrombectomy devices of different generations. A previous study reported that several factors may contribute to short recanalization times, such as an experienced stroke team, rational prehospital logistics, and emergency room management (40). In our center, a diagnostic and interventional neuroradiology service and anesthesia team are available 24 h per day, 7 days per week.

Inconsistent with the results of this trial, a previous study demonstrated that patients with AIS undergoing MT had worse clinical outcomes when treated with GA compared with CS. The reasons are complicated (41). First, delay in treatment initiation, particularly in the GA group, has been hypothesized to be a plausible explanation. A survey of SVIN members also found that surgeons felt the most important limitation of GA was time delay (37, 42). In the current trial, there was no significant difference between the two groups with respect to time metrics. The reason may be due to the highly specialized anesthesia care team, better visualization of the clot, and fewer pauses in the GA group. Our results also demonstrate that a well-organized workflow is associated with no delay in performing GA for MT, and there is no effect on outcomes compared to CS. A previous study found that the outcome of CS patients at discharge was mainly dependent on the NIHSS score at presentation, post-treatment mTICI score, and a history of transient ischemic attack. However, there were no significant between-group differences in such factors in our trial. Another factor that contributes to worse clinical outcomes in GA patients is hemodynamic changes (43). A previous study found that tracheal intubation and extubation could provoke coughing reflexes that raised intrathoracic and intracranial pressure, reducing cerebral blood flow and blood supply to the penumbra (44). In our trial, more stable hemodynamics were recorded in the CS group. However, functional outcomes and mortality rate at discharge and 3 months after stroke were comparable between the two groups. The reason may be due to the fewer DBP fluctuations in our study (45). A meta-analysis also suggested that close monitoring and strictly controlling hemodynamics seem more important regardless of the choice of agents and anesthetic technique (21). Previous study also suggest that the relationship between arterial pressure and outcomes of AIS is a U-shaped curve, with the lowest risk of death and disability occurring at a systolic arterial pressure of 150 mm Hg. Additionally, cerebral blood flow becomes linearly dependent on cerebral perfusion pressure because of the loss of cerebrovascular autoregulation (46). It is usually thought that arterial pressure should be monitored carefully to avoid a drastic reduction, and a reduction after recanalization should be allowed for by the neurointerventionalists and neurologists to avoid potential hemorrhagic conversion. Consistent with the results of previous research, considerable fluctuations in hemodynamics occurred in our trial even if CS patients received only DEX and propofol (30). Previous study reported that the cumulative dose of norepinephrine was independently associated with poor outcome. The reason may be that vasopressors could only improve peripheral blood pressure without improving blood flow to the ischemic penumbra (47). In our trial, the vasopressor phenylephrine use increased in the GA group, although there was no clinical significance.

Some intravenous and inhalational anesthetic agents used for GA are known to be associated with hypotension and are independent predictors of poor neurological outcomes in the acute phase of AIS and during the endovascular procedure of MT (48). To eliminate the interference of inhalational anesthetic agents, we adopted total intravenous anesthesia in this trial. Total intravenous anesthesia may also affect the outcomes, as the various related intravenous drugs have different neurochemical, neurophysiologic, and systemic effects. Animal research has found that inhaled anesthetics inhibit neuronal apoptosis and reductions in astroglial processes, reducing glial scarring. They can also enhance inhibitory synaptic transmission by increasing γ-aminobutyric acid and glycine levels, inhibiting excitatory *N*-methyl-d-aspartate–type glutamate and neuronal nicotinic acetylcholine receptors, activating K2P channels, and causing K^+^ channels to leak (49). However, previous research has also demonstrated that both inhalational and intravenous anesthetic agents can profoundly reduce cerebral blood flow and take blood flow from ischemic areas with poor autoregulation; the stress ultimately led to cerebral hypoperfusion and exacerbation of the ischemic injury (48). Dexmedetomidine affects the blood pressure in a dose-dependent manner (27). Nichols et al. (50) divided CS into four levels (no sedation, mild sedation, heavy sedation, and pharmacologic paralysis) and found that male sex and no or mild sedation were independently related to a good outcome; heavy sedation and pharmacological paralysis were independent predictors of mortality. As a result, we used mild sedation in the CS group in this trial. There was no significant between-group difference in the site of vascular occlusion in our trial, although a previous study reported that the site of vascular occlusion influences outcomes.

A previous study reported end-tidal carbon dioxide (ETCO_2_) values at 60 and 90 min during surgery instead of the minimum and maximum ETCO_2_ associated with outcomes. This may be due to hypocapnia-induced cerebral decreases in cerebral blood flow promoting the transformation of the ischemic penumbra into irreversibly infarcted tissue (51). Although hypercapnia increased cerebral perfusion and had a neuroprotective effect after transient global cerebral injury in animal models, a previous study found that the regional cerebral vasodilatory response to hypercapnia may be impaired in patients with symptomatic cerebral ischemia (52). As a result, we kept ETCO_2_ between 35 and 40 mm Hg in our study.

Because of the relatively high incidence of coexisting coronary artery disease, hypertension, and diabetes mellitus, patients undergoing MT are at high risk of other complications. The results of previous study found that using NIHSS scores alone in multivariate models may not capture some important aspects of stroke severity (53). As a result, patients were recruited according to both NIHSS and ASA scores in our study. Infarct volume, which is a strong predictor of functional outcomes in patients with AIS, was improved to different extents in the two groups at 48 h post-intervention, although there was no significant between-group difference in infarct volume. Consistent with previous research, most procedure-related complications in this trial were due to the thrombolytic effect, infarct size, or device vessel incompatibility rather than patient movement (54). The main success parameter of a sedation protocol is the rate of conversion to GA. Only 9.52% patients in the CS group needed to convert to GA because of hypoxemia in our trial, which is lower than the rates in previous studies (SIESTA: 14.3%; GOLIATH: 15.6%) (19, 24). Previous studies have reported that patients who converted to GA on an emergency basis did not develop more complications, which is consistent with the results in this trial. However, other studies found that emergency conversion to GA could result in hypoxia, time delay, and aspiration and increased risk of hypotension and pneumonia (30, 32). As a result, it is reasonable to favor CS during MT for anterior AIS-LVO only if immediate conversion to GA is possible. In agreement with the results of the SIESTA and ANSTROKE trials, we found a higher rate of pneumonia in the GA group (19, 25).

Our study has several limitations. First, hemodynamic variables were documented at 3-min intervals, and it is possible that significant hemodynamic fluctuations were missed in the periods between the assessments. Second, regional differences in anesthetic practice may exist. The trial was performed under the guidance of a highly specialized anesthesia care team in our center. Many centers without dedicated anesthesia teams may take longer to induce anesthesia and may not manage hypotension rapidly (55). Third, because of the relatively small sample size in this trial, the results need to be confirmed in a larger multicenter randomized controlled study. Fourth, although serum glucose regulation is very important for promoting neurological recovery in AIS patients, we did not record the perioperative blood sugar level, as the goal of post-procedural management of glucose is to keep blood sugar at 70–140 mg/dL in our center. Further studies should examine the relationship between glucose levels and clinical outcomes after AIS. Finally, transcranial color Doppler (TCCD) ultrasound is widely used for real-time monitoring of cerebral hemodynamic conditions (56). However, we did not adopt TCCD ultrasound during the whole treatment for both technical and economic reasons.

In summary, in this single-center study, anesthetic management with GA or CS during MT had no differential impact on the functional outcomes and mortality rate at discharge or 3 months after stroke in patients with AIS, although more stable hemodynamics and a lower incidence of pneumonia were recorded in the CS group.

## Data Availability Statement

The datasets analyzed in this article are not publicly available. Requests to access the datasets should be directed to 25758860@qq.com.

## Ethics Statement

The studies involving human participants were reviewed and approved by Institutional Review Board of Liaocheng People's hospital. The patients/participants provided their written informed consent to participate in this study. We obtained approval from the institutional review board of our hospital (ethics committee of Liaocheng People's Hospital, chairman: Dawei Wang) for this randomized clinical trial. The study was also registered at chictr.org (ChiCTR-IPR-16008494). Informed consent was obtained from each patient's representative.

## Author Contributions

CR, YL, GX, GL, and JG conceived and designed the trail. CR analyzed the data. YL, GX, GL, and JG collected the data. GX, CR, GL, YL, JW, and JG wrote this paper.

### Conflict of Interest

The authors declare that the research was conducted in the absence of any commercial or financial relationships that could be construed as a potential conflict of interest.
